# Boost Tendon/Ligament Repair With Biomimetic and Smart Cellular Constructs

**DOI:** 10.3389/fbioe.2021.726041

**Published:** 2021-08-31

**Authors:** Jianping Zhao, Xiang Wang, Jinyu Han, Yin Yu, Fei Chen, Jun Yao

**Affiliations:** ^1^ Department of Orthopedics Trauma and Hand Surgery & Guangxi Key Laboratory of Regenerative Medicine, International Joint Laboratory on Regeneration of Bone and Soft Tissue, The First Affiliated Hospital of Guangxi Medical University, Nanning, China; ^2^ Center for Materials Synthetic Biology, Institute of Synthetic Biology, Shenzhen Institute of Advanced Technology, Chinese Academy of Sciences, Shenzhen, China

**Keywords:** tendon/ligament repair, biomimetic, smart hydrogels, anisotropic adhesion, gene therapy

## Abstract

Tendon and ligament are soft connective tissues that play essential roles in transmitting forces from muscle to bone or bone to bone. Despite significant progress made in the field of ligament and tendon regeneration over the past decades, many strategies struggle to recapitulate basic structure-function criteria of native ligament/tendon. The goal here is to provide a fundamental understanding of the structure and composition of ligament/tendon and highlight few key challenges in functional regeneration of these connective tissues. The remainder of the review will examine several biomaterials strategies including biomimetic scaffold with non-linear mechanical behavior, hydrogel patch with anisotropic adhesion and gene-activated scaffold for interactive healing of tendon/ligament. Finally, emerging technologies and research avenues are suggested that have the potential to enhance treatment outcomes of tendon/ligament injuries.

## Introduction

### Basic Anatomy of Tendon/Ligament

#### Composition

Tendon and ligament are crucial components of the musculoskeletal system that serve important mechanical roles in providing joint stability and enabling mobility. The primary difference between these connective tissues is what they connect: tendon connects muscle to bone and transmits forces from muscle to bones while ligament form links between bones. Despite of the differences in functions, tendon and ligament are composed of similar constituents that include collagen (roughly 70–80% dry weight), proteoglycans, elastic fibers and other minor proteins, and water (∼65–70% wet weight). Many different types of collagens have been found in tendon and ligament, including collagen I, II, III, IV, XI and XIV. Collagen I is the most predominant type in both tissues, and ligament also contain 9–12% of collagen Ⅲ (O’brien, 1997). Other collagens are involved in regulating fibril and fiber assembly, such as Collagen Ⅱ is detected at enthesis which is the connective tissue between tendon or ligament and bone (O’brien, 1997; Lu and Thomopoulos, 2013).

#### Hierarchical Structure

**FIGURE 1:**
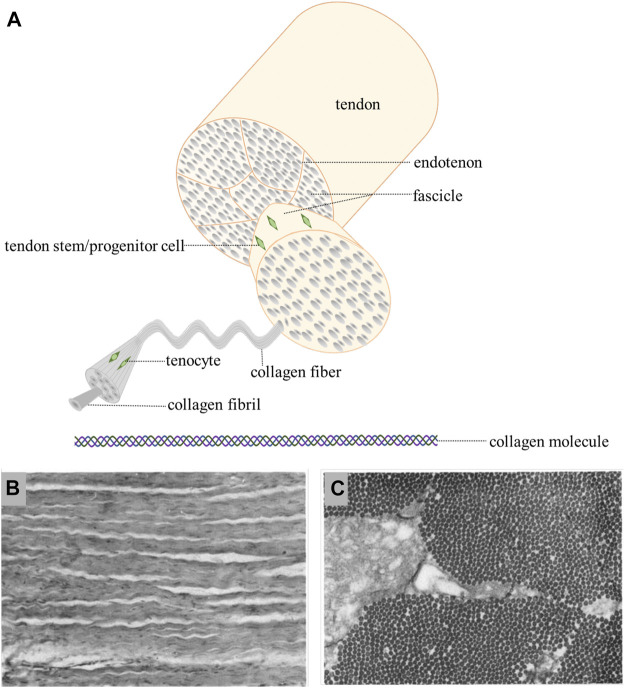
**(A)**: hierarchical structure of tendon/ligament [Adapted from No et al. (2020)] (Created with BioRender.com). **(B)**: longitudinal section show crimped and uniformly oriented fibers (Strocchi et al., 1991). **(C)**: in transverse section, the same size collagen fibrils are seen (Strocchi et al., 1991).

Tendon and ligament are hierarchical structures that build from the nanoscale to the centimeter scale, mainly composed of type I collagen. Soluble tropocollagen molecules produced by tenocytes and ligamentocytes form insoluble collagen molecules by crosslinking, which subsequently aggregate into microfibrils and progressively form the microscopically visible collagen fibrils. Collagen fibers, the basic units of tendon and ligament, are composed of bundles of fibrils. Subsequently, collagen fibers bind to a fine layer of connective tissue surrounding them, namely endotenon and aggregate into the primary fiber bundles (subfascicles), which then form the secondary fiber bundles (fascicles). Tertiary bundles are composed of these fascicles, which eventually make up the tendon and ligament (Figures 1A–C) (Kannus, 2000; Reese et al., 2013). The orientation of collagen fibers depends on the tension to which they are subjected. Most collagen fibers are longitudinal and parallel to the tendon axis, while there are a few collagen fibers that are horizontal and transversal to transmit forces from both directions (Jozsa et al., 1991). In microscopic level, the structures of collagen fibers and fibrils are crimped and wavy (Hochleitner et al., 2018). The crimp angles are determined by anatomical location of tendon and ligament, such as the mean crimp angle in rectus femoris tendon, vastus intermedius tendon and patellar ligament of rat, which is 141.5 ± 15.0°, 122.3 ± 14.8° and 146.2 ± 12.2° respectively (Franchi et al., 2009). In an ovine model, several parameters are significantly different between tendon and ligament. For example, the diameters of ovine tendon are larger than those of ligament (Rumian et al., 2007). The stress-strain curves of tendon and ligament usually are sigmoidal shaped, consisting of the initial “toe region”, the linear response region, the “yield point” and the final ultimate load. The ultimate load is an important parameter in mechanical assessment of different tendon and ligament. It is generally believed that the ultimate load of tendon is greater than that of ligament (Birch et al., 2013).

Besides, the discrepancies in mechanical properties between human and animal models shouldn’t be ignored and we listed the mechanical properties of T/L in most investigated models which include human, goat, rats, rabbits and (Table 1).

**TABLE 1:** Mechanical properties of tendon/ligament in different species.

Tendon/ligament	Ultimate stress (MPa)	Ultimate strain (%)	Young’s modulus (MPa)
Human Achilles Wren et al. (2001)	86 ± 24	9.9 ± 1.9	822 ± 211
Human rotator cuff Carpenter et al. (2005)	45.1 ± 19.6	0.18 ± 0.13	629 ± 230
Human ACL Chandrashekar et al. (2006)	24.36 ± 9.38	0.28 ± 0.07	113 ± 45
Rat Achilles Volper et al. (2015)	11.6 ± 1.7	17.7 ± 2.0	136 ± 18.9
Rabbit Achilles Yamamoto et al. (2017)	44.9 ± 4.6	-	494.0 ± 38.6
Rabbit ACL Woo et al. (1992)	60 ± 8	-	516 ± 64
Rabbit subscapularis tendon Grumet et al. (2009)	8.2	0.16	56
Ovine extensor digital tendon Sun et al. (2020)	89.76 ± 15.40	40.01 ± 10.12	574.20 ± 253.81

#### Cellular Composition and Molecular Cues

In addition to extracellular matrix (ECM) components, tendon and ligament contain cells, mainly fibroblasts but also progenitor cells. Tenocytes are the major cell types in tendon, while in ligament, ligament fibroblasts are the predominant cells, which are surrounded by matrix and only represent a small percentage of the ligament volume. Communication among these dispersed fibroblasts may be achieved by cytoplasmic extensions (Frank, 2004).

There are three major transcription factors involved in the growth, development and homeostasis regulation of tendons and ligaments: Scleraxis (Scx), Mohawk (Mkx), early growth response factor1 (Egr1). Sck expressed in tendon progenitor cells is responsible for regulating early tendon growth and development. It has been known that the transcription of Col1a1, Col1a2, Acan and Tnmd is Sck-dependent. In contrast to Sck, Mkx works primarily during tendon maturation (Asahara et al., 2017). As Dochevaa et al. reviewed, there are several growth factors participate in the repair of tendon/ligament, including bone morphogenetic proteins (BMP), connective tissue growth factor (CTGF), insulin-like growth factor-1(IGF-1), basic fibroblast growth factor (bFGF), platelet-derived growth factor (PDGF), vascular endothelial growth factor (VEGF) and transforming growth factor beta (TGFβ) (Docheva et al., 2015), which promote the synthesis of collagen, glycosaminoglycan and other ECM, improve cell proliferation and angiogenesis, change the way of tendon healing, and enhance their mechanical properties.

### Damage, Native and Clinical Repair of Tendon/Ligament

The development of tendonopathy (tendinopathy) is related to many factors, such as age, sex, weight, overuse, fatigue, and occupational environment. Tendonopathy is usually divided into chronic injury and acute injury according to the pathology and can be classified into tendonitis (tendinitis) and tendonosis (tendinosis) by pathogenesis (Docheva et al., 2015). Tendonitis is an inflammatory infiltration of the primary affected tendon and is often used clinically to describe a specific clinical syndrome. Tendonosis is not a disease that must have symptoms. Its essence is not an inflammatory disease but a degenerative disease (Maffulli, 1998). Unlike normal white tendon with a firm fibrous texture, tendon with chronic tendon injuries are yellowish-brown, thin, and brittle. With histological staining, tendinopathy shows thinner collagen fibers, increased extracellular matrix, increased cell death, altered cellular structure, and more new blood vessels (Kaux et al., 2011). Acute tendon injuries usually refer to tendon ruptures caused by acute trauma and spontaneous tendon ruptures due to tendinopathy, which has been proven to be the primary cause of tendon ruptures (Sharma and Maffulli, 2005). Similarly, ligament injuries can be caused by various internal and external factors, including trauma, overuse, poor tissue structure or growth and development. In a case of violent trauma, it is more common that more than one ligament gets injured, whereas the rest of the musculoskeletal system may also get involved (Rodrigues et al., 2013).

**FIGURE 2:**
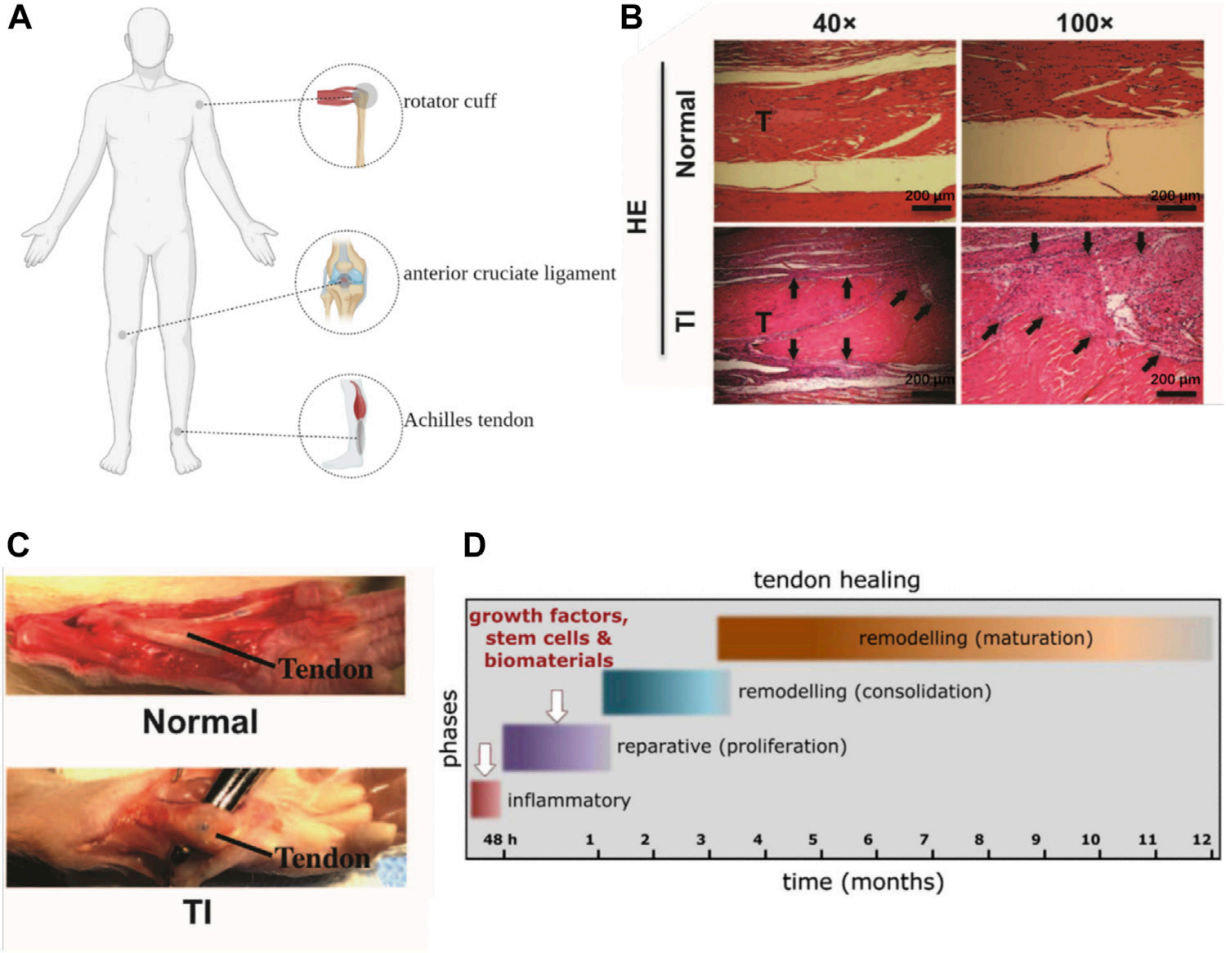
**(A)**: Anatomical position of Achilles tendon, rotator cuff and anterior cruciate ligament (Created with BioRender.com). **(B, C)**: General view and HE staining of tendon injury (TI) (scar and adhesion) and normal control (Chen et al., 2017). **(D)**: the process of tendon repair (Docheva et al., 2015).

The native repair process of ligament is similar with that of tendon, thus we will only briefly describe the repair process of tendon here. There are two different ways of tendon repair. One occurs intrinsically which involves the epitenon and endotenon tenocytes. The other develops extrinsically taking cells from surrounding sheath and synovium. Tendon healing is a complex process that require the cooperation of cells, ECM, cytokines, and other proteins (Sharma and Maffulli, 2005). The process is usually described as four overlapping sequential stages: inflammatory stage, proliferation stage, remodeling stage and maturation stage (Figure 2D). At the onset of injuries, hematomas form and pro-inflammatory cytokines are produced by mast cells which recruit neutrophils, macrophages and monocytes to the injury site. Angiogenesis is induced by the increased secretion of vascular endothelial growth factor (VEGF). ECM, primarily collagen III, are subsequently synthesized by the recruited fibroblasts at the injury site which is a hallmark of proliferation stage. During the remodeling stage, ECM production and cell activities are decreased. Collagen III is replaced by collagen I and the collagen fibers start to organize along the direction of tendon stress. At the stage of maturation, the crosslinking of collagen fibrils increase and mature tendonous tissues form with their mechanical strength being gradually improved (Docheva et al., 2015). However, due to the lack of appropriate mechanical stimulation, scar tissue formation as well as other reasons, the naturally repaired tendon can hardly restore their full functions and the risk of re-rupture of these repaired tendon remains high.

Among different tendon and ligament, Achilles tendon (Raikin et al., 2013), anterior cruciate ligament (Grassi et al., 2020) and rotator cuff (Tashjian, 2012) showed prominently higher incidents of rupture, which aroused wide interests in the repair of these tissues. And these three kinds of tendon and ligament are particularly important for the body to maintain normal physiological function, it is necessary to choose the appropriate treatment to repair the tendon and ligament to the maximum extent (Figure 2A). The selection of repair methods for tendon and ligament diseases in clinical practice depends on the severity and the site of diseases as well as the age of patients. For tendinopathy or mild to moderate tendon and ligament injuries with incomplete ruptures and good blood supply of peritendinous tissue, conservative treatment is generally adopted (Orava and Kujala, 2005). Common conservative treatments include rest, non-steroidal anti-inflammatory drugs, local hormonal blocking, immobilization, laser treatment and bracing (Lim et al., 2019). However, some studies have found that immobilization during conservative treatment will attenuate therapeutic effects, resulting in decrease of mechanical strength of repaired tendons, such as ultimate tensile and deviation characteristics. Appropriate rehabilitation training can reduce adhesion and promote flexor tendon healing (Voleti et al., 2012; Barfod, 2014). Surgical treatments are required when conservative treatments failed and patients suffer from severe tendon/ligament injuries such as a complete tendon rupture or multiple ligament injuries and need a rapid restoration (Lim et al., 2019). For massive rotator cuff tears, Thorsness R et al. proposed to treat repairable tissue with superior capsular reconstruction in patients who were young or did not have significant arthritis. Reverse Total Shoulder Arthroplasty is preferred by elderly patients with pseudo paresis, tendinopathy and low repair needs (Thorsness and Romeo, 2016). In some special cases, such as the defect or shortening of tendon and ligament, autograft, allograft and xenograft may be introduced (Sakabe and Sakai, 2011). The application of these tissue transplant in musculoskeletal system repair is promising. Nevertheless, the success rate of autograft is low and complications such as pain, instability and mechanical incompetence can hardly be ignored. Besides, general concerns like immunological rejection and zoonosis in allograft and xenograft should be considered when therapies are selected (Lim et al., 2019).

As has been mentioned, existing clinical treatments can rarely restore the full functions of tendons and ligaments. The major limitations of the conservative treatment are poor healing, scarring and the risk of refracture. The main histological difference between native tendon/ligament and scar tissue is that former fiber orientation is more disordered than the latter (Bruns et al., 2000). Besides, from a biomechanical properties perspective, scar tissue exhibited decreased strength, increased brittleness and was more likely to induce adhesion formation compared with native tissue (Voleti et al., 2012). Scar tissue does not match the mechanical properties of native tissue, nor does it have the nonlinear response mode of tendon/ligament tissue. Therefore, the repaired tissue is prone to re-fracture when it is subjected to a force less than the stress limit of normal tissue (Nourissat et al., 2015). Similarly, surgical treatment also has complications such as scar healing. Postoperative infection and postoperative adhesion are also problems that cannot be ignored in surgical treatment. The occurrence of adhesions is related to the factors that include tissue injury and surgical procedures. It prolongs the healing time and a second operation is often required. Improper selected suture materials, incorrect suture methods, premature postoperative rehabilitation training and surgical anatomical location are the main reasons leading to poor surgical treatment effect (Voleti et al., 2012) (Figures 2B,C). Due to above limitations, researchers are exploring different functional tissue substitutes to promote regeneration and healing of tendon and ligament tissue.

## Smart Hydrogels to Address Some Key Challenges of Tendon/Ligament Regeneration

Tissue Engineered Medical Products (TEMPs) are a series of medical products designed by tissue engineering principle that can repair, improve and regenerate the structure and function of tissues and organs (Wang et al., 2016). The potential of TEMPs in tendon/ligament repair has been extensively studied over the past two decades. Products were developed to improve the natural healing process of tendon or ligament and to enhance their mechanical properties during surgical repair. Tendon and ligament are dense and anisotropic connective tissues, which contains collagen fibers in response to mechanical stimulation, cellular components regulating ECM and a large volume of water that ensures elasticity (Rodrigues et al., 2013). To mimic natural tendon or ligament, a tissue-engineered tendon or ligament need to meet the following requirements. First, TEMPs should have good biocompatibility that allows for cell adhesion, proliferation, migration, extracellular matrix deposition. Second, such products should have mechanical properties equivalent to those of normal tissues.

In this review, we focus on emerging strategies for the design and implementation of TEMPs to promote tendon/ligament repair, we highlight few strategies which we believe could greatly boost tendon/ligament repair in the future including: 1) biomimetic fibrous scaffold which mimic the unique biomechanical properties of tendon/ligament, 2) Bioinspired hydrogel patch with anisotropic surface adhesion to reduce postoperative adhesion and 3) biomaterial-assisted gene therapy for in-site drug delivery to facilitate tendon/ligament repair (Law et al., 2016).

### Fibrous Scaffolds With Non-Linear Mechanical Behavior to Mimic the Extracellular Matrix of Native Tissue

The native human tendon/ligament primarily consists of aligned crimped collagen I fibers ranging from 1 to 30 um in diameter grouped into subfascicles. These organized and aligned collagen fibrils are critical for the generation and direction of force by providing the topographical cues to the imbedded cells. Experimentally, stress-strain curves of native tendon/ligament exhibit a J-shaped and a ‘toe region’, where the stress on the tissue is minimal typically at strain values lower than 2–5%. Above this ‘toe region’ follows a linear reaction region. This non-linear stress-strain behavior is critical for tendons and ligaments in resisting cyclic fatigue and effects of creep, and protect the collagen fibers and other ECM from the effects of low physiological strain (No et al., 2020). It is thus envisioned that fibrous scaffold with biomimetic geometry as native tendon/ligament will offer promising results for tendon/ligament regeneration.

**FIGURE 3:**
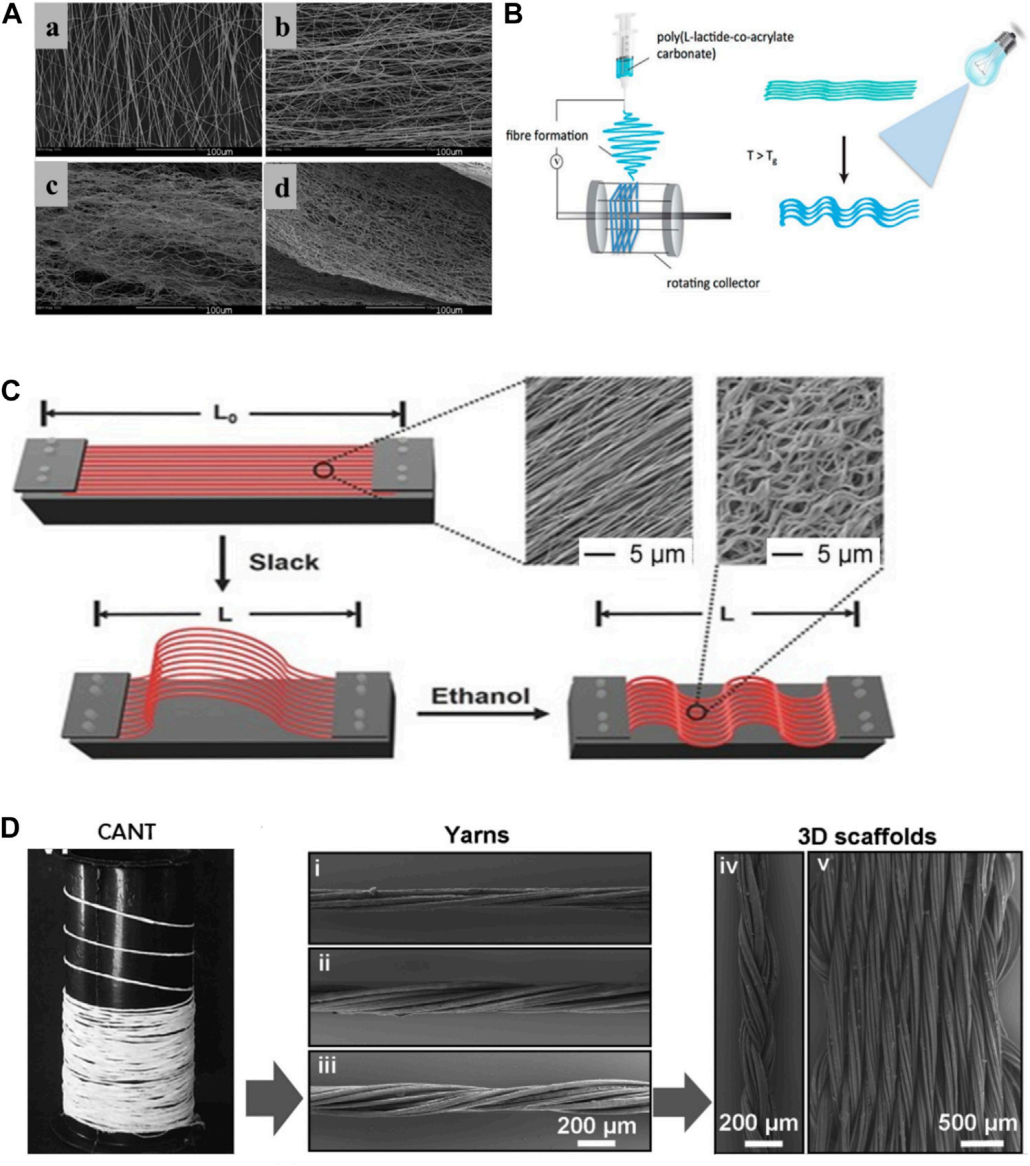
**(A)**: a)control, b)incubated at 4°C, c)incubated at 22°C, d)incubated at 37°C (Surrao et al., 2010). **(B)**: fabrications of crimped and cross-linked fiber scaffolds (Chen et al., 2014). **(C)**: schematic diagram of controllable crimp fiber (Liu et al., 2015). **(D)**: the 3D structure of the scaffold was obtained by braiding (Laranjeira et al., 2017).

Over the years, Electrospinning has been most commonly adapted technique to constructing scaffold with specific size (0.1–1.5 um) and pattern after a charged polymer solution is extrude from a needle onto a static or rotating collector (No et al., 2020). Surrao et al. synthesized self-crimping nanofibers by putting dimethylformamide (DMF) plasticized poly (L-lactide-co-D, L-lactide) (PLDLLA) electrospun nanofiber films into PBS solution at a temperature higher than its glass transition temperature. The post-spinning thermal treatments induced the formation of crimped morphology of fabricated nanofibers which resembles the pattern of anterior cruciate ligament. The crimp parameters (amplitude and wavelength) of the nanofibers were controllable by changing the difference between the operating temperature and the glass transition temperature of fibers (Surrao et al., 2010) (Figure 3A).

Dynamic loading on aligned fibrous scaffold has been shown to further enhance secretion of tendon/ligament specific ECM components (No et al., 2020). Amsden et al. explored the effects of different mechanical stimulation amplitude on crimped or non-crimped nanofiber membrane and found that 10% strain amplitude could significantly increase the synthesis of ECM in crimped fibers compared to straight fibers (Surrao et al., 2012). However, crimped nanofibers synthesized in previous studies are prone to lose their microstructures after dynamic stimulations. Chen et al. synthesized a crimp-stabilized fibrous scaffold by electrospinning and photo-cross-linking the copolymerized L-lactide with acryloyl carbonate (AC) monomer. Cross-linking stabilized the crimped morphology of scaffolds which possess a Young’s modulus comparable to that of human anterior cruciate ligament (Chen et al., 2014) (Figure 3B).

Crimped fibrous scaffolds produced by electrospinning are usually too dense which cells can hardly infiltrate through. To better control the crimp pattern of fibrous scaffold, Liu et al. electrospun arrays of uniaxially aligned poly (lactic acid) (PLA) filaments with an initial length (L_0_). The stretched filaments were then socked in ethanol and shrank into short crimped filaments with a new length (L). By controlling the ratio of L/L_0_, nanofibers with different degree of crimping were made. Their results showed that fibers with a L/L_0_ ratio of 50% maintained highest tendon fibroblast viability (Liu et al., 2015) (Figure 3C). To obtain a scaffold with higher porosity, Spencer et al. co-electrospun poly (ethylene oxide) (PEO) and an water-insoluble material, followed by water washing to remove PEO. This method enlarges the spacing between the nanofibers and increases the available space for the fibers to shrink, thus providing a better non-linear mechanical response than those treated with traditional thermal treatment alone (Szczesny et al., 2017).

Compared with two-dimensional scaffolds, three-dimensional porous scaffolds improve cell permeability and nutrient exchange. Laranjeira et al. used electrospinning technology to construct continuous aligned nanofiber threads by combining PCL, chitosan and cellulose nanocrystals, and weaved the two-dimensional nanofiber threads into a three-dimensional structure through different textile processes. The braided three-dimensional scaffold not only had the non-linear stress-strain curve similar to the normal tendon tissue, but also induced the tenogenic-like differentiation of adipose stem cells, and formed the neotissue similar to native tendon tissue (Laranjeira et al., 2017) (Figure 3D).

In summary, most crimped fibers synthesized in above studies can meet the requirements for human ligament/tendon repair, in consideration of the Young’s modulus of native human ligament/tendon under physiological conditions (Table 2). A controllable crimped fiber was constructed by Surrao et al., but its Young’ modulus was low and failed to meet the lowest limit of normal value (Surrao et al., 2010). This shortage was overcome in subsequent studies by stimulating extracellular matrix differentiation with dynamic loading (Surrao et al., 2012), modifying composition and cross-linking (Chen et al., 2014), incorporating new monomers (Liu et al., 2015; Szczesny et al., 2017), or manufacturing with braided technology (Laranjeira et al., 2017). These crimped fibers with non-linear response have better biomechanical properties, which pave the way for future exploration of biomimetic scaffold for engineered T/L tissue.

**TABLE 2:** Summary of mechanical properties of crimped fibers in literature.

Composite	Ultimate stress (MPa)	Ultimate strain (%)	Young’s modulus (MPa)
PLDLLA Surrao et al. (2010)	0.193 ± 0.044	-	0.349 ± 0.069
PLDLLA Surrao et al. (2012)	-	-	33 ± 2^a^
			17 ± 1^b^
			8.7 ± 0.4^c^
P (LLA-AC) (12%AC) Chen et al. (2014)	-	-	86 ± 5 (dry uncross-linked)
			222 ± 28 (dry cross-linked)
			26 ± 1.4 (hydrated cross-linked)
PLLA/PEO Szczesny et al. (2017)	-	-	3.0 ± 1.0
PCL/chitosan/cellulose nanocrystals Laranjeira et al. (2017)	-	111 ± 20 (yarn 6)	-
		105 ± 10 (yarn 9)	
		135 ± 10 (yarn 12)	
		48 ± 12 (braided)	
		104 ± 50 (woven)	

PLDLLA Surrao et al. (2012).

a mechanically stimulated crimp-like cultures.

b mechanically stimulated uncrimped cultures.

c static cultures.

However, despite some advantages of synthetic polymers over natural polymers, few limitations of synthetic polymers do exist. For instance, the degradation time of synthetic polymers are normally much longer than the time for the formation of regenerative tissue and thus results in mechanical irritation and chronic inflammation. In addition, many synthetic polymer materials will evoke host response and even fibrous encapsulation occurs in the long time. Therefore, an ideal scaffold should not only possess the appropriate biomechanical properties but also biological functions to recreate a pro-regenerative microenvironment for tendon/ligament repair.

### Bioinspired Hydrogel Patch for Tendon/Ligament Repair

**FIGURE 4:**
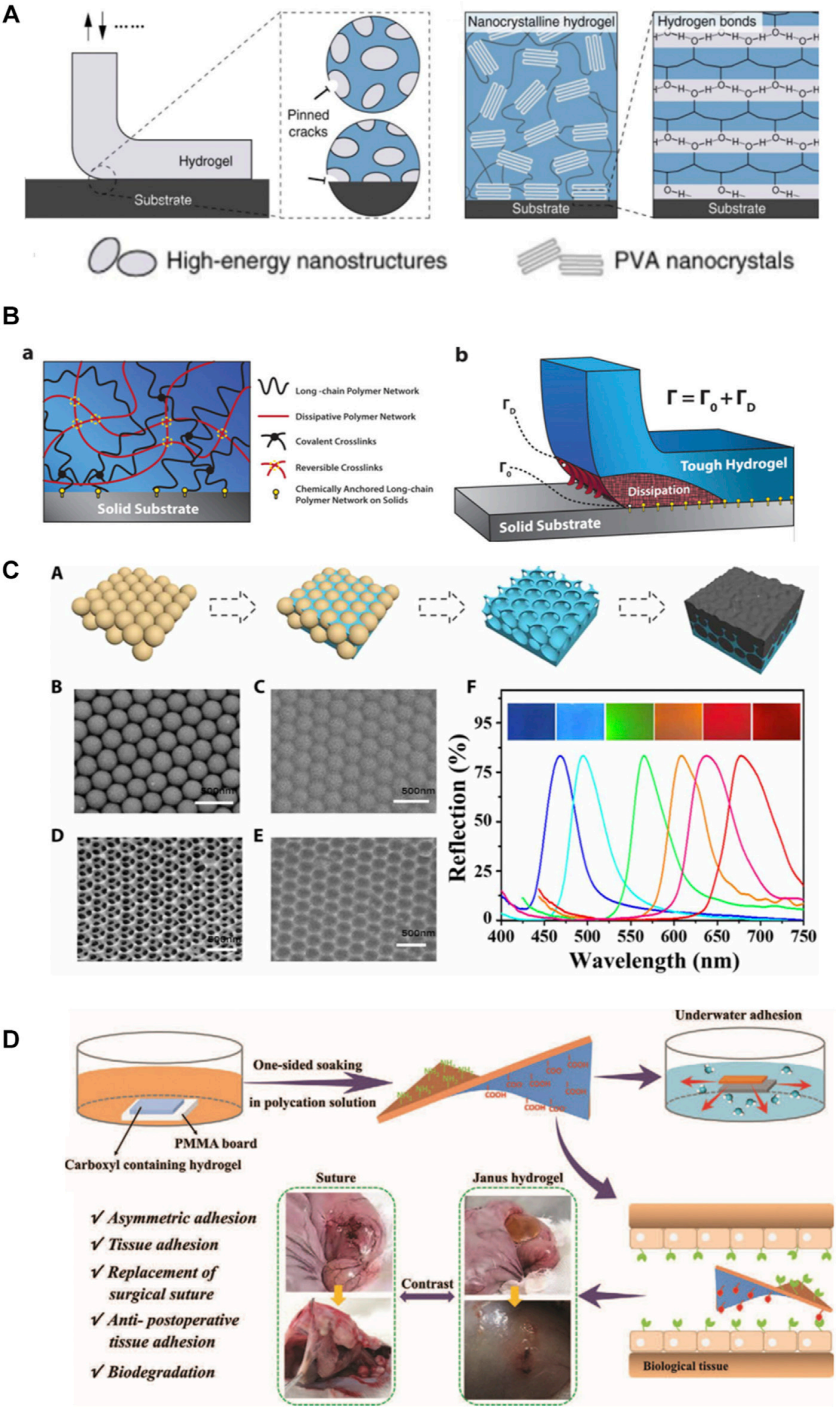
**(A)**: high-energy nanostructures bioinspired fatigue-resistant adhesion, fatigue-resistant adhesion of poly (vinyl alcohol) (PVA) hydrogel (Liu et al., 2020). **(B)**: design idea of hydrogel adhesion to different material surface (Yuk et al., 2016). **(C)**: the illustration depicts the formation of a bioinspired structural color patch (Wang et al., 2020a). **(D)**: the illustration depicts the process of construction of a Janus hydrogel and repair of rabbit’s perforated stomach (Cui et al., 2020).

Although surgical reconstruction is the primary treatment for tendon and ligament related injuries, many individuals suffer from postoperative complications including re-rapture, reduced function and postsurgical adhesion between adjacent tissues. Bioadhesives are widely used in the repair of skin (Chen et al., 2018), cartilage (Ozturk et al., 2020), muscle (Hong et al., 2019) and other tissues due to their excellent tissue adhesion and cytocompatibility. Numerous bioadhesives have been reported thus far, for instance, adhesive hydrogels were made of gelatin methacryloyl and methacryloyl-substituted recombinant human tropoelastin by cross-linking under visible light (Annabi et al., 2017). Strong adhesion hydrogels using a combination of physical and chemical reactions with an adhesion interface and background hysteresis have been explored (Li et al., 2017). Gan et al. constructed a highly adhesive hydrogel with biocompatibility, long-term adhesion and anti-infection ability by using Ag-lignin nanoparticles with the reversible conversion ability of quinone-catechol (Gan et al., 2019). There are also physiological adhesion sites in tendon/ligament tissues, which are the tendon-bone interface connecting tendon/ligament with bone or cartilage. Among them, the calcified fibrochondral regions are the key to fatigue-resistant adhesion. In order to meet the normal physiological needs, the interface toughness of some cartilage-bone interfaces should meet the requirements of 800Jm−2 after 1 million loads per year (Liu et al., 2020). Adhesive can be used as an adjuvant for surgical treatment of tendon/ligament injuries, with attention to anisotropy and the direction of force applied. The toughness of the adhesive will also have a large impact on the treatment efficacy (Avgoulas et al., 2019). Nanocrystalline domains composed of PVA hydrogels were anchored to solid substrates through annealing treatment, endowing fatigue-resistant adhesion to engineering materials including glass, stainless steel and ceramics. When applied between articular cartilage and rigid machines, the hydrogel coating exhibits excellent wear and slip resistance (Liu et al., 2020) (Figure 4A) researchers also anchored hydrogels to a solid substrate through silane modification and EDC chemistry, and confirmed that interfacial toughness is superior to the adhesions between tendon-bone interfaces (Yuk et al., 2016) (Figure 4B). Adhesive hydrogel can also be used as Photochemical Tissue Bonding (PTB) as a supplement to surgical sutures. Chan et al. tested the PTB treatment of Achilles tendon ruptures in rats. Rose Bengal (RB) solution was applied to the ruptured end of Achilles tendon and irradiated with laser to obtain a PTB treated model. PTB was found as an effective approach facilitating tendon repair (Chan et al., 2005). However, the traditionally used (green) laser (532 nm) has poor tissue penetration, which requires a direct exposure to wounds. To address this issue, RB-containing Chitosan/β-GP hydrogels were loaded with upercoversion nanoparticles which covert the infrared light (808 nm) to the green light. Noninvasive photochemical sealing was achieved for Achilles tendon ruptures, which was even more effective than the conventional PTB (Zhu et al., 2020).

Postoperative adhesion between tendon/ligament and surrounding tissue is a unmet clinical need, it is thus of clinical significance to design and construct a bioadhesive that can adhere injured tendon/ligament and facilitate healing while prevent post-surgical tissue adhesion simultaneously (Zhang et al., 2021). Studies have shown that the external repair through the surrounding tendon sheath and synovial origin cells is the main cause of adhesion, and the association between inflammatory response and adhesion should not be underestimated (Zhou and Lu, 2021). Various types of hydrogels, including hyaluronic acid, have been used to repair tendons and ligaments in order to reduce extrinsic healing and inflammation (Zhang et al., 2021). They can adhere to different tissue surfaces, simulate the function of extracellular matrix, allow cells, cytokines, oxygen and other components penetrate, and play a physical barrier to reduce the extrinsic repair of tendons and inflammation, thus promoting the functional repair of tendon and ligament.

Therefore, ideal hydrogels for tendon and ligament repair should not only meet the requirement of stable and fatigue-resistant adhesion to the tissue surface, but more importantly, reduce the adhesion between the damaged tissue and the surrounding tissue. Yan et al. constructed a double-layer adhesive hydrogel patch, with the outer layer of which was a hydrophobic anti-inflammatory and anti-adhesive PLGA electrospun nanofilm containing ibuprofen, and the inner layer was a PEG-PLV hydrogel containing basic fibroblast growth factor. The researchers found that this unique drug delivery system was able to effectively deliver the drug *in situ* over a long period of time, reducing adhesion and thus promoting Achilles tendon healing in rats (Yan et al., 2021). By enclosing B-type synovial cells in self-assembled peptide hydrogel and adding electrospun PCL nanofilm on the outer layer, which physically insulates tendon tissue from subcutaneous tissue contact, Imere et al. successfully constructed a system that provides moderate sliding in early healing and can resist peripheral adhesion (Imere et al., 2021). Despite the great potential and increasing interest in these anisotropic adhesive hydrogels, their applications thus far have been limited to monitoring cardiac activity (Wang et al., 2020a) (Figure 4C), preventing postoperative adhesion of the abdominal wall, and suturing gastric tissue (Cui et al., 2020) (Figure 4D). To the best of our knowledge, no anisotropic adhesives have been used in load-bearing tissues such as tendon/ligament defects but the potential of this smart hydrogel adhesive for tendon/ligament defects remains attractive and promising.

### Biomaterial-Assisted Gene Therapy to Treat Tendon/Ligament Defects

Growth factors can be produced and acted at every stage of tendon/ligament healing, especially in the early stage of healing, and play a pivotal role in inducing cell growth and tissue development (Docheva et al., 2015). However, traditional *in-situ* loading of growth factors around tissues is often difficult and full of challenges. It is not only necessary to control the timing of the release of growth factors, but also to control the amount of release. Responsive drug delivery system solves some of the issue of growth factor delivery but the high cost and short half-life of growth factors still limits their wide application in tendon/ligament repair in clinic. Gene therapy offers an exciting approach to improving tendon and ligament healing (Docheva et al., 2015). Gene therapy was defined by The Food and Drug Administration (FDA) as “the administration of genetic material to modify or manipulate the expression of a gene product or to alter the biological properties of living cells for therapeutic use.” Sustained and regulated expression of transgenes *in situ* can be achieved by gene therapy and this regulatory manner can enhance the biological activities of proteins of interest (Evans and Huard, 2015). A variety of growth factors, signal molecules, anti-inflammatory factors can be synthesized *in situ* through gene therapy (Ilaltdinov et al., 2020).

According to the central dogma of molecular biology, the transmission of genetic information is a gradual process from DNA to RNA and finally to proteins. Genetic engineering is a technology that modifies and processes many different kinds of DNA and RNA. These two nucleic acids have their advantages and disadvantages when used. DNA has the risk of integration into the host genome, while RNA can avoid the risk but its transfection efficiency is limited and it is easy to be cleared (Attia et al., 2021). Foreign gene transfer can be introduced into cells either by viruses or non-viral vector such as cationic liposomes vectors. The former makes use of viral innate ability of infection to delivery genes into recipient cells and is engineered non-replicable. Adenoviruses, lentiviruses and retroviral related viruses are the most commonly used viruses in gene therapy. Because viral vectors are often complex and expensive, non-viral vectors with high safety, low restriction and good immunogenicity are often more attractive (Docheva et al., 2015). Genes can be delivered into host tissue *via in vivo* (*in vivo* injection) or *ex vivo* methods (*in vitro* transduction). *In vivo* delivery can be divided into two ways: the use of vectors alone and the combination of a matrix. The *ex vivo* strategy of gene delivery through autologous cells or allogeneic cells has attracted more and more attention because it can not only artificially regulate gene expression accurately but also promote cell proliferation (Evans and Huard, 2015).

Using a recombinant adenovirus transduction method, the researchers successfully transferred the target gene LacZ expressing *E. coli* β-galactosidase into the tendon and tendon sheath of chickens, and demonstrated that the gene expression product can be maintained for a long time without degradation (Lou et al., 1996). In order to investigate the relationship between intracellular focal adhesion kinase (FAK)-related signaling pathway and tendon adhesion, Lou et al. transfected the recombinant adenovirus containing the FAK gene (PP125 FAK) into tenocytes *in vitro* and into tendons of chickens *in vivo*, and successfully observed the overexpression of proteins and adhesion (Lou et al., 1997). However, due to the immunogenicity of adenovirus vectors to a certain extent, adenovirus-associated vectors have been used in most subsequent studies. Moreover, Zhu et al. demonstrated that compared with plasmid vector, the tendon healing induced by adenovirus-associated vector is most consistent with the normal tendon healing process, so this vector may be a more suitable vector for gene transfer in tendons (Bei et al., 2006). Through the transduction of basic fibroblast growth factor (bFGF) by adeno-associated virus vector, Wang and colleagues studied the effect of gene therapy on the regulation of collagen fibers by tenocytes *in vitro* (Xiao et al., 2005), Tang and colleagues explored the effect of gene therapy on flexor tendon healing *in vivo* (Jin et al., 2008). Combined with previous studies on bFGF *in vivo* and vascular endothelial growth factor (VEGF) *in vitro* induction of tenocytes, the researchers respectively loaded the two genes into adeno-associated viruses, and found that in the condition of no increased adhesion or decreased gliding, tendon repair showed a two-fold increase in strength and less adhesion to surrounding tissue (Tang et al., 2016).

Non-viral vectors have gained increasing attention because of their superior safety, low immunogenicity and minimal risk of viral genes being integrated into host cells. Chemically modified mRNAs that encode bFGF are more stable and less immunogenic than unmodified mRNAs, and can enhance repair when directly injected into defective tendon tissue (Herbst et al., 2018). Given the traditional use of non-viral vectors, which are usually injected into tissues as a solution, the anchoring is often uncontrollable. Therefore, researchers chose to fix the non-viral vector to the scaffold material before implantation *in vivo*, and then anchor the scaffold material to the wound area, so as to realize *in situ* gene therapy (Zaitseva et al., 2018). Using degradable PLGA nanoparticles to encapsulate plasmids carrying bFGF and VEGF genes, Zhou’s team demonstrated the effectiveness of transfection by conducting *in vivo* tendon and *in vitro* tenocyte experiments (Yang et al., 2018). Zhou et al. coated the cyclooxygenase-engineered miRNA (COX-1 and COX-2) plasmids in PLGA nanoparticles and then placed the nanoparticles in a HA hydrogel. They proved this system not only reduced inflammation level by downregulating significantly COX-1 and COX-2 expression, but also decreased formation of adhesion between tendon and the surround (Zhou et al., 2018).

**FIGURE 5:**
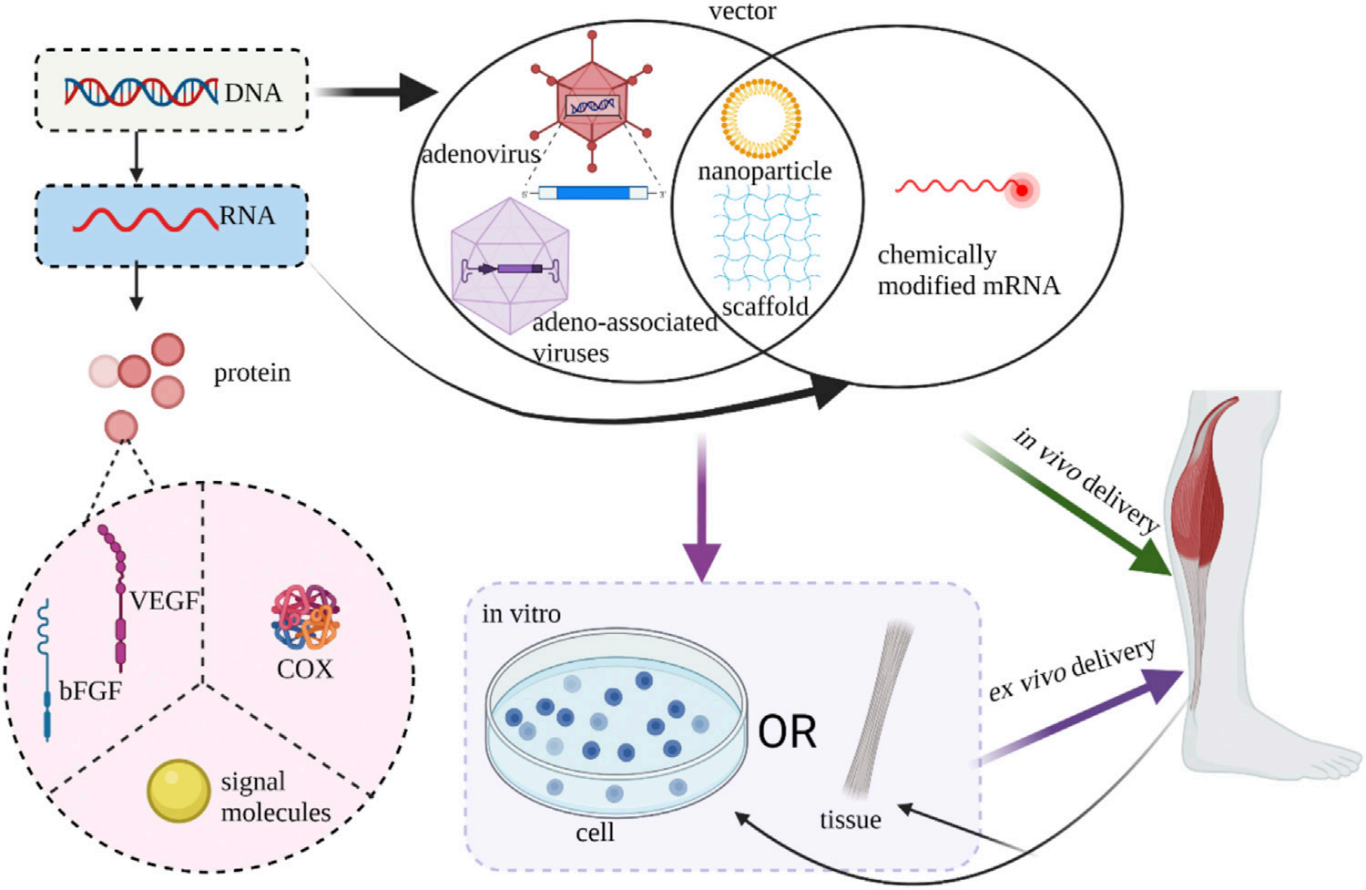
Current strategies of gene therapy of tendon/ligament (Created with BioRender.com).

Different from the repair of common tendon and ligament injuries, attention should be paid to the unique fibronochondral transitional zone of the tendon in the repair of the tendon and bone connection interface, so greater mechanical strength and appropriate cartilaginous development environment should be met. Lattermann et al. successfully introduced the target gene into the tendon-bone interface using an adenovirus vector (Lattermann et al., 2004). Smad3 small interfering RNA (Wang et al., 2020b), SOX9 gene (Zhu et al., 2014), BMP4 gene (Coen et al., 2011), COX2 gene (Rundle et al., 2014), MT1-MMP gene (Gulotta et al., 2010) and BMP-2 gene (Martinek et al., 2002) can promote the strengthening of tendon-bone junction healing and early physiological repair through adenovirus or lentivirus transduction (Figure 5).

The results of gene therapy for tendons/ligaments are encouraging, but there is a lack of extensive experimental confirmation of optimal nucleic acids, carrier types, and delivery modes. Based on the successful application of functional genetic circuit in immunotherapy and other cell therapy in recent years (Nims et al., 2021), we envision that living materials constructed by synthetic biology methods including gene therapy will further promote the development of tendon/ligament repair.

## Summary and Future Perspectives

In this review, biomechanical, hierarchical structure and cellular composition of native tendon and ligament were briefly discussed, followed by introduction of biomechanical and biological problems facing repair and healing of tendon and ligament injuries in native and clinical scenario. There have been tremendous improvements to clinical treatment options for tendon or ligament injuries, however, many issues still remain. We highlight few advanced engineering strategies aimed to restore the properties of healing tissue to normal levels. These strategies include designing fibrous scaffold to mimic the ultrastructural and biomechanical properties of native tendon or ligament, hydrogel adhesives to facilitate the injured tissue healing while prevent the post-operational adhesion between tissues. In addition, biomaterial-assisted gene therapy and genome editing tools offer an exciting approach to improving tendon and ligament healing as it could be more precisely regulate the gene transcription during tendon/ligament healing thus is potential to increase the therapeutic efficacy in clinical application. However, lack of efficient delivery strategies to the target cells and tissues, such as ligament and tendons which have dense extracellular impedes the widespread use of genome editing technology in clinic. These new therapies will likely be approved on a case-by-case basis as they need to be rigorously validated in cells, animals, and in humans for safety. Despite of these challenges, the potential of genome editing and advanced gene therapy technology in the development of new treatment strategies is confidently expected to have a major effect on the practice of tendon/ligament repair.
